# Is nitrogen-modified atmosphere packaging a tool for retention of volatile terpenes and cannabinoids in stored *Cannabis sativa* inflorescence?

**DOI:** 10.1186/s42238-024-00253-9

**Published:** 2024-12-20

**Authors:** Luke L. MacLaughlin, Mason T. MacDonald

**Affiliations:** Department of Plant, Food, and Environmental Science, Faculty of Agriculture, Dalhousie University, Truro, NS B2N 5E3 Canada

**Keywords:** Aromatics, Cannabigerol, High value compounds, Monoterpenes, β-Myrcene, Sesquiterpenes, Δ^9^-Tetrahydrocannabinol, Terpenoid

## Abstract

Modified atmosphere packaging (MAP) alters the gaseous composition of air surrounding packaged goods to prevent deleterious oxidation associated reactions. MAP has been adopted for the storage of cannabis, though a recent study revealed little difference in terpene content under MAP conditions. Questions regarding its efficacy for preservation of high value compounds like terpenes and cannabinoids lost during postharvest storage remain. The goal of this research is to determine weather N_2_ MAP preserves high value compounds of cannabis during its postharvest storage. This experiment followed a completed randomized block design. There were two factors of interest. The first was storage atmosphere (atmospheric or N_2_ MAP). The second was storage duration (18, 46, or 74 days). The experiment was then blocked by cannabis chemovar using 5 different chemovars. The concentration of 17 cannabinoids was evaluated through UPLC-UV and 61 volatile terpene compounds through GC–MS. Concentrations were compared over time and between storage treatments. There were no significant differences in total cannabinoids and volatile terpene compounds over time or between storage treatments. Individual cannabinoids Δ^9^-THC, CBG, CBNA, CBC, THCV, and THCVA all increased during storage time while THCA decreased. CBG and THCV only increased under MAP storage. Individual aromatics limonene, β-pinene, α-pinene, camphene, and terpinolene all only decreased during storage under N_2_ MAP. Only caryophyllene oxide and α-humulene increased under N_2_ MAP storage. β-Myrcene decreased under atmospheric storage, but not under N_2_ MAP. While N_2_ MAP had no effect on the preservation of total cannabinoids and aromatics during storage, it did influence several individual compounds. CBG, THCV, and α-humulene all increased under N_2_ MAP. N2 MAP also maintained the concentration β-myrcene over time, though the preservation of β-myrcene was offset by a decrease limonene. Overall, N_2_ MAP was not needed for preservation of most high value compounds but did have an effect of some compounds with reputed therapeutic benefits.

**Supplementary Information:**

The online version contains supplementary material available at 10.1186/s42238-024-00253-9.

## Introduction

Cannabis (*Cannabis sativa*) originated near central Asia more than 10,000 years ago and would eventually be established throughout the world via anthropogenic means, with fossils dating its human consumption back thousands of years (Pisanti and Bifulco 2018; Okazaki et al. 2011). Cannabis is typically recognized for its content of cannabinoids, terpenes, terpenoids, flavonoids, and alkaloids (Kollar et al. 2016). Recent research has emerged demonstrating the importance of terpenes and terpenoids for perceived product quality (Booth and Bohlmann 2019; Tanney et al. 2021; Plumb et al. 2022) and their potential medical benefits are well characterized (Miyazawa and Yamafuji 2005; Gaggiotti et al. 2020; Hanŭs and Hod 2020). Yet volatile terpenes and terpenoids contributing to cannabis aroma decrease up to 50% within 1 month of postharvest storage (Bueno et al. 2020), and degradation of cannabinoids is well documented (Fairbairn et al. 1976; Grafström et al. 2019; Lindholst 2010; Mazzetti et al. 2020). Industry interest retaining these high-value compounds (HVCs) has seen the adoption of modified atmospheric packaging (MAP) technologies within the Canadian cannabis market space.

MAP modifies the gaseous composition of atmospheric air within a sealed container to extend shelf life and preserve product quality (Utama 2020). Degradation of lipids via oxidization is often associated with reductions in product flavour and aroma (Labuza and Dugan 1971; Aj 1996). Oxygen molecules present in the air react with unsaturated fatty acids, ultimately forming unstable hyperoxide free radicals that undergo a cascade of further reactions (Domínguez et al. 2019). Most cannabinoids, terpenes, and other high-value metabolites are stored in the glandular trichomes of cannabis (Johnson 1975; Hammond and Mahlberg 1978; Kim and Mahlberg 1997; Livingston et al. 2019). Structurally, the trichome is encapsulated by a cuticle which includes varying lipidic constituents (Mahlberg and Kim 1991; Lara et al. 2015; Liu et al. 2022) and its oxidation represents a potential mechanism for accelerated loss of HVCs under atmospheric storage conditions (MacLaughlin and MacDonald 2024). Several studies have demonstrated the efficacy of MAP for delaying lipid oxidation, improving food product stability, and extending shelf life of other products (Marasca et al. 2016; Zhao et al. 1994; Kitabayashi et al. 2018).

MAP infrastructure represents a significant cost to producers, and limited research on its efficacy for storage of dried cannabis currently exists (MacLaughlin and MacDonald 2024). Bueno et al. (2020) recently reported no improvement in the retention of volatile terpenes over an atmospheric control with similar argon-based MAP strategies for storage of dried cannabis. However, N_2_ MAP technologies have demonstratable efficacy for preservation of volatiles across a range of other agriculture products that include coffee (*Coffea arabica)*, lemon verbena (*Aloysia citrodora*), potato chip seasoning, and milk powder (Marin et al. 2008, Ebadi et al. 2016, Agarwal et al. 2018; Lloyd et al. 2009).

The composition of cannabinoids and terpenes is affected by many factors, both endogenous and exogenous, which makes analysis a challenging task. Cannabis cultivars are well established to have vast differences in cannabinoid profiles (Hazekamp et al. 2016, Danziger and Bernstein 2021). For example, average tetrahydrocannabinol (THC) from control “Fuji” cultivars was approximately 60–70% higher than “Himalaya” cultivar (Danziger and Bernstein 2021). The positioning of inflorescences, height of the tissue, and type of tissue sampled all also have a significant effect in chemical profiles (Bernstein et al. 2019a, Danziger and Bernstein 2021, Ghosh et al. 2023). Cultivation practices can also have a profound effect on the chemical profile of cannabis. Growing cannabis at a higher plant density decreased several cannabinoids from lower inflorescences, though had much less effect on high inflorescences (Danziger and Bernstein 2022). Even mineral nutrition or other soil additives can affect cannabinoids. Supplementation with NPK fertilizer increased cannabigerol (CBG) concentration of flowers by 71% and decreased cannabinol (CBN) in flowers and inflorescences (Bernstein et al. 2019b). Further, the ratio of N:P:K fertilization could introduce variability. Supplementation of P at rates greater than 5 mg L^−1^ decreased the concentration of Δ^9^-THC and cannabidolic acid (CBDA) (Shiponi and Bernstein 2021). Supplementation with K decreased the acidic forms of cannabinoids while non-acidic forms were generally unaffected (Saloner and Bernstein 2022). Similarly, terpenoids also tended to decrease with increasing concentrations of K, though this effect had a degree of genotype specificity (Saloner and Bernstein 2022). Considering the myriad of well-established factors above, any chemical profile analysis of cannabis must take great care to minimize the experimental error caused by spatial variability or growing conditions.

This study aims to compare changes in HVCs of dried cannabis inflorescence stored under N_2_ MAP and atmospheric conditions, addressing limitations of previous work by increasing sampling size, developing a standardized sampling protocol to reduce phenotypical variance, and offer comprehensive insights on MAP of dried cannabis for preservation of HVCs. The results of this study will enable producers to make informed decisions regarding implementation of MAP infrastructure and exploring potential options for improved consumer experiences.

## Methods

### Growing conditions

Five cannabis (*Cannabis sativa* L.) cultivars (“Mango Sour”, “Apple Pancakes”, “CV3”, “Chem Dozer”, and “Blackwater”) were grown at a health Canada licensed facility (EastCann, Halifax, NS, Canada). Rooted cuttings were individually transplanted into 8 L Canna COCO flex bags (Canna Canada, Ottawa, ON, Canada) and grown under LED lighting throughout both photoperiod phases. Vegetative growth was carried out under a 24-h lighting schedule for 42 days, followed by flowering with a 12/12-h photoperiod for 67 days. Lower branches were removed at day 1 of flowering, and defoliation occurred at day 28 of flowering yielding a slightly modified version of the “bottom branches and leaves removal” training method described by (Danziger and Bernstein 2021). Plants were grown for a combined total of 95 days and harvested by hand harvested for drying.

### Experimental design

**Fig.1:**
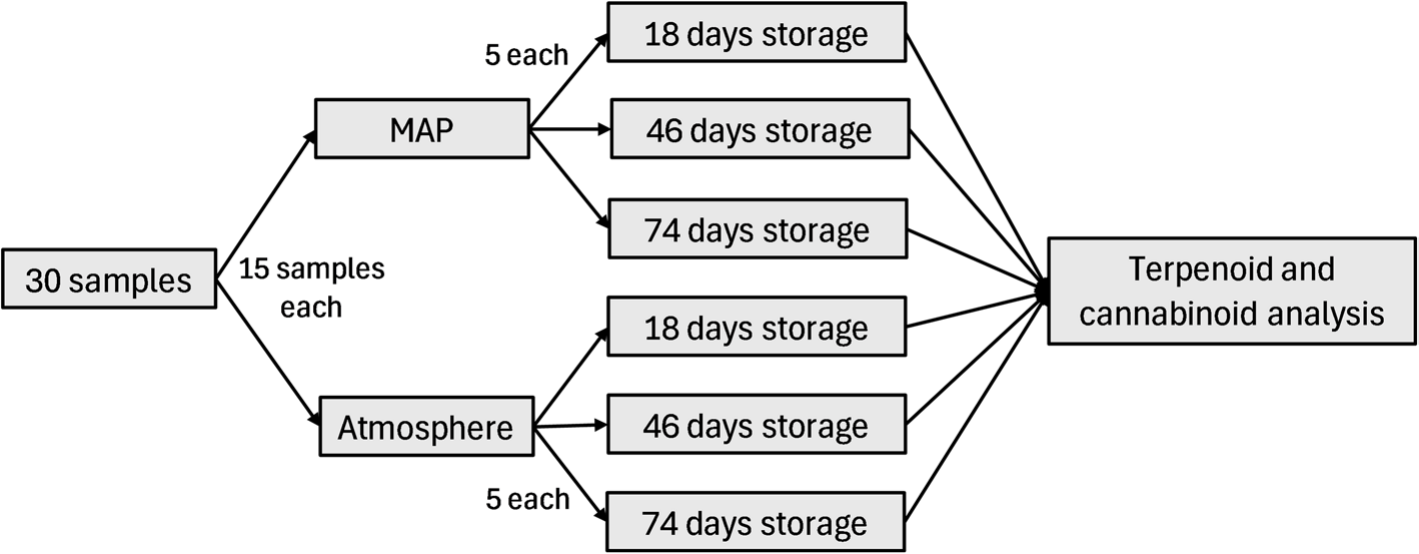
Experimental design for analysis for MAP and storage duration on terpenoids and cannabinoids in cannabis

This experiment followed a completely randomized block design with two factors of interest and a potential interaction effect (Fig. 1). The first factor was MAP. Treated cans underwent active MAP treatment via a proprietary canning line utilizing liquid nitrogen to achieve oxygen levels ≈4% (N_2_ MAP) while the control cans were sealed under atmospheric air at oxygen levels ≈ 21% (atmospheric storage). The second factor was storage time, where samples were under storage for 18 days, 46 days, and 74 days. Samples had to be shipped for analysis preventing analysis prior to storage. However, it is reasonable to assume that there were no significant differences initially because there would be no degradation yet and MAP treatment would not have been imposed. Finally, the design used cultivar as a blocking factor. Ultimately, this experiment required 30 samples.

### Sample collection

Two apical inflorescences of approximately the same height and size were selected per plant to produce an A and B sample and reduce phenotypical variance, while also limiting variation from positioning. Height and type of tissue have significant variation in chemical profiles (Bernstein et al. 2019a, Danziger and Bernstein 2021), which would make a true comparison impossible without carefully selected samples. Samples were carefully labeled to identify the individual plant as well as the A and B inflorescence.

### Postharvest treatment

Labeled samples were hung and air dried at 14 °C and 50% relative humidity for 14 days, then hand trimmed. Samples of dried inflorescence were collected from the apical end of branches and placed into steel cans (48.0 mm in height and 83.3 mm in diameter), until a sample weight of 3.5 g was obtained. Treatment (N_2_ MAP) or no treatment (atmospheric conditions) was randomly assigned to the A inflorescence, with B receiving the remaining option. Samples were shipped to Supra Research and Development (Kelowna, BC, Canada) for analysis. Response variables quantified include15 different major cannabinoids and 23 terpenes. Analyzed cannabinoids were Δ^9^-THC, Δ^8^-THC, CBC, CBCA, CBDA, CBDV, CBDVA, CBG, CBGA, CBL, CBN, CBNA, THCA, THCV, and THCVA. Analyzed terpenes and related compounds were α-Pinene, terpinolene, camphene, linalool, β-pinene, β-myrcene, β, caryophyllene, α-humulene, limonene, *trans*-nerolidol, eucalyptol, guaiol, fenchone, *cis*-β-ocimene, *trans*-β-ocimene, caryophyllene oxide, octyl acetate, borneol, α-terpineol, α-bisabolol, terpinen-4-ol, geranyl acetate, and β-cedrene. Additional details can be found in Supplemental Tables I and II).

### Cannabinoid analysis

Extraction and analysis of cannabinoids was conducted according to Riordan-Short (2023a). Dried inflorescence samples were ground with an electric grinder until homogenous and subsamples prepared for respective analytical methodologies. A 0.2 g subsample was weighed and extracted using 20 mL methanol. Samples were placed in an ultrasonic bath and sonicated for 10 min, vortexed, and then cold stabilized at −20 °C for 1 h. Samples were then centrifuged for 5 min at 4200 rpm. A 10 µL aliquot of supernatant was diluted in 990 µL of mobile phase diluent mix and vortexed for 10 s.

Cannabinoid analysis was conducted via a Vanquish™ UPLC with UV-detector (Thermo Fisher Scientific, Waltham, MA, USA) and an Ascentis™ Express 90 Å C18 15 cm × 2.1 mm × 2 µm (Supelco #50,814-U) UPLC column. Mobile phase and instrument details are shown in Supplemental Table III. A 40 µg/mL standard stock solution was made consisting of all tested cannabinoids, then a calibration curve was made with 0 (blank), 0.01, 0.05, 0.1, 0.5, 2, 10, and 25 µg/mL dilutions. A 1 mg/mL ibuprofen solution was used as a check standard. Also, a second cannabinoid solution made from reagents with a different lot number than those use in calibration was used as an independent check standard. All standards were purchased from Restek (Bellefonte, PA, USA).

### Terpenoid analysis

Extraction and analysis of terpenoids was conducted according to Riordan-Short (2023b). A 0.2 g subsample was collected and extracted in 20 mL of hexane. Samples were vortexed for 10 s, then placed in an ultrasonic bath for 10 min. Samples were then vortexed again for 10 s and centrifuged for 5 min at 4200 rpm. A 10 µL aliquot of supernatant was diluted into 990µL of hexane.

Terpenoid analysis was completed with an ISQ™ 7000 single quadrupole GC–MS and TRACE™ 1310 column (Thermo Fisher Scientific, Waltham, MA, USA). Specific instrument parameters are described in Supplemental Tables IV and V. Standards were prepared by mixing 5 µL of Terpene Mega Mix #1 and #2 (Restek, Bellefonte, PA, USA) in 1240 µL of isopropanol and toluene, respectively. A 10 µg/mL solution of *d*_*3*_-Linalool was used as an internal standard. The calibration curve was made with 0 (blank), 0.04, 0.08, 0.16, 0.32, 0.63, 0.125, 0.25, 0.5, and 1.0 µg/mL dilutions. Also, a second terpenoid solution made from reagents with a different lot number than those use in calibration was used as an independent check standard.

### Statistical analysis

Data analysis was conducted using Minitab® 21.4.2 statistical software. A general linear model was used with the model including main effect of atmospheric storage, main effect of storage duration, the interaction between atmospheric storage and storage duration, and the blocking effect of chemovar. Factor levels included chemovar with 5 levels, time with 3 levels, and treatment with 2 levels and 29 total degrees of freedom for each response. Response variables analyzed included 15 separate cannabinoids, total cannabinoids, 22 terpenes, 1 aromatic compound, and total aromatic compounds for a total of 40 response variables. Fisher’s LSD pairwise comparisons with 95% confidence were then conducted for all response variables. Since the chemovar had no interactive effect with any other variable, response variable concentrations of all compounds were analyzed and reported. Statistical assumptions of normality and constant variance were confirmed through Minitab. Independence is assured through randomization and the fact unique samples are used for each storage duration versus repeated measurements on the same sample.

## Results

There was no significant difference due to MAP treatment or storage time for total cannabinoids (*p* = 0.226). Individual cannabinoids with significant differences (*p* < 0.05) over time included THCA, Δ^9^-THC, CBG, CBNA, CBC, THCV, and THCVA (Table 1). All following cannabinoid comparisons are between 18 and 74 days. There was a 216% increase in Δ^9^-THC between in the atmospheric treatment, but significantly higher 359% increase under N_2_ MAP. There was no change in CBG under atmospheric storage, but 49% increase in CBG under N_2_ MAP. There was a 17–20% increase in CBNA during storage, but no difference between MAP and atmosphere treatments. CBC increased during storage but there was no difference between MAP and atmospheric treatments. Finally, THCV did not change over time under atmospheric storage, but increased significantly under N_2_ MAP. Consequently, N_2_ MAP had higher Δ^9^-THC, THCVA, and THCV after storage than the atmospheric control. THCA was the only cannabinoid to significantly (*p* < 0.05) decrease, albeit only by 6–7% and with no difference between atmospheric and MAP treatments.

**Table 1:** Mean cannabinoid concentrations of cannabis stored in ambient atmosphere or N_2_ modified atmospheric packaging. Cannabinoids that differ significantly (*p* < 0.05) after storage or between treatments are marked with an asterisk. Those means with different superscript letters are significantly different as determined by Fisher’s least significant difference and indicate differences across each row. The Δ column indicates the direction of change between 18 and 74 days when it was significant

Compound (mg/g)	Atmospheric Storage				N_2_ MAP Storage			
	18 days	46 days	74 days	Δ	18 days	46 days	74 days	Δ
THCA	197.7^A^	187.1^BC^	185.3^C^	-	203.4^A^	190.4^B^	189.4^BC^	-
Δ^9^-THC *	9.3^D^	20.4^C^	29.4^B^	+	8.2^D^	19.6^C^	37.6^A^	+
THCVA*	16.92^AB^	16.29^AB^	15.02^B^		16.61^AB^	16.71^AB^	18.82^A^	
CBGA	6.66	6.22	7.29		6.44	6.52	7.31	
CBCA	1.77	1.89	2.07		2.00	1.92	2.22	
CBG *	0.75^AB^	0.75^AB^	0.75^AB^		0.63^B^	0.81^AB^	0.94^A^	+
CBNA *	0.59^BC^	0.57^C^	0.71^A^	+	0.58^C^	0.59^C^	0.68^AB^	+
CBDA	0.46	0.57	0.61		0.48	0.58	0.63	
CBC *	0.02^C^	0.57^AB^	0.57^AB^	+	0.00^C^	0.46^B^	0.65^A^	+
THCV *	0.11^AB^	0.11^AB^	0.11^AB^		0.00^B^	0.11^AB^	0.36^A^	+
CBN	0.02	0.11	0.23		0.00	0.11	0.23	
d8-THC	0.00	0.11	0.12		0.00	0.23	0.15	
CBDV	0.14	0.00	0.00		0.00	0.00	0.00	
CBDVA	0.00	0.11	0.00		0.00	0.00	0.00	
CBL	0.00	0.00	0.00		0.11	0.00	0.00	
Total *	234.2^B^	234.7^B^	242.0^AB^		238.7^AB^	238.1^AB^	258.8^A^	

There was no significant difference due to MAP treatment or storage time for total terpenoids concentration (*p* = 0.191 and 0.174, respectively). However, there were significant differences (*p* < 0.05) in many specific terpenoids (Table 2). Some compounds only changed over time under one treatment. β-myrcene was the most prominent terpenoid in cannabis but decreased 33% under atmospheric storage with no significant decrease under N_2_ MAP. Conversely, limonene, β-pinene, α-pinene, camphene, and terpinolene all only decreased under N_2_ MAP. Caryophyllene oxide and α-humulene only increased under N_2_ MAP storage by 29% and 52%, respectively while fenchol increased by 16% under atmospheric storage. Other compounds, such as α-terpineol and *trans*-nerolidol increased over time regardless of storage treatment.

**Table 2:** Mean terpenoid and related compound concentrations of cannabis stored in ambient atmosphere or N_2_ modified atmospheric packaging. Means that differ significantly (*P* < 0.05) after storage or between treatments are marked with an asterisk. Those means with different superscript letters are significantly different as determined by Fisher’s least significant difference and indicate difference across each row. The Δ column indicates the direction of change between days 18 and 74 when it was significant

Compound (µg/g)	Atmospheric Storage				N_2_ MAP Storage			
	18 days	46 days	74 days	Δ	18 days	46 days	74 days	Δ
β-Myrcene*	10,004^A^	8,294^AB^	6,724^B^	-	10,457^A^	8,602^AB^	8,392^AB^	
β-Caryophyllene*	4,230^AB^	5,749^AB^	5,124^AB^		4,198^B^	5,969^A^	5,482^AB^	
Limonene*	4,860^AB^	4,339^B^	4,239^B^		5,428^A^	4,519^AB^	4,048^B^	-
α-Humulene*	1,439^B^	1,725^B^	1,596^B^		1,443^B^	1,784^B^	2,187^A^	+
Linalool*	1,090^B^	1,849^A^	1,275^B^		1,177^B^	1,446^AB^	1,448^AB^	
α-Bisabolol	622	763	623		581	680	701	
β-Pinene*	688^AB^	716^AB^	610^BC^		761^A^	742^A^	595^C^	-
Fenchol*	383^C^	514^A^	446^AB^	+	431^BC^	503^AB^	463^AB^	
α-Pinene*	370^BC^	431^AB^	361^BC^		437^AB^	452^A^	345^C^	-
α-Terpineol*	312^B^	419^A^	409^A^	+	349^B^	408^A^	421^A^	+
*trans*-Nerolidol*	276^C^	363^B^	384^A^	+	290^BC^	361^B^	422^A^	+
Guaiol	146	203	215		178	188	208	
Borneol	218	199	155		230	201	158	
*trans*-β-Ocimene*	182^AB^	150^B^	154^B^		238^A^	187^AB^	189^AB^	
Fenchone	82	92	398		115	114	69	
Caryophyllene oxide*	119^B^	162^A^	147^AB^		123^B^	151^AB^	159^A^	+
Camphene*	105^AB^	123^A^	110^AB^		118^AB^	122^AB^	97^B^	-
Terpinolene*	70^AB^	69^AB^	39^B^		92^A^	92^A^	46^B^	-
*cis*-β-Ocimene	25	29	20		29	30	28	
Geranyl acetate*	0^B^	87^A^	13^B^		0^B^	65^A^	0^B^	
Terpinen-4-ol	0	23	9		0	23	0	
Octyl acetate	0	23	9		0	23	0	
β-Cedrene*	0^C^	19^A^	3^C^		0^C^	19^A^	0^C^	
Total Terpenoids	25,221	26,341	23,063		26,675	26,681	25,458	

There were two significant (*p* < 0.005) trends over storage time that occurred when compounds were grouped into terpene and terpenoid classes (Table 3). The first is that monoterpenes decreased 62% more under N_2_ MAP than atmospheric storage. The second is that sesquiterpenes increased 50% more under N_2_ MAP than atmospheric storage. Changes under storage resulted in a shift in monoterpene:sesquiterpene from 2.01 under atmospheric storage to 1.79 under N2 MAP. There were no significant differences in the relative changes in terpenoid classes.

**Table 3:** Percent change for each class of aromatic compounds between 18 and 74 days after atmospheric or N_2_ MAP storage. Percentages were determined as the total of all individual analytes belonging to each classification. The Δ column represents N_2_ Map (%) – Atmospheric (%). All classes had a significant change over time, regardless of storage treatment. Those classes marked with * represent significant differences (*p* < 0.05) in the Δ between storage treatments

Class	Atmospheric (%)	N_2_ MAP (%)	Δ
Terpenes	−82	−94	−12
Monoterpenes*	−114	−176	−62
Sesquiterpenes*	32	82	50
Terpenoids	104	97	7
Monoterpenoids	65	51	−14
Sesquiterpenoids	39	46	7

## Discussion

Cannabinoids and aromatic compounds of dried cannabis inflorescence are HVCs with important roles in cannabis’ effects (Taura et al. 2007) and consumer perception of quality (Booth and Bohlmann 2019; Tanney et al. 2021; Plumb et al. 2022). A single month of storage can see a 50% loss in levels of terpenes (Bueno et al. 2020), and degradation of cannabinoids via various pathways over time has been consistently demonstrated (Fairbairn et al. 1976; Grafström et al. 2019; Lindholst 2010; Mazzetti et al. 2020). In our study, a canning line employing active N_2_ MAP under current commercial application was investigated for its efficacy to improve retention of cannabis HVCs during postharvest storage.

To our knowledge this paper represents the only modern study on changes in cannabinoids over time in plant material of dried cannabis under N_2_ MAP since the work of Turner et al. (1973), who reported the absence of light as more important than N_2_ for maintaining levels of Δ^9^-THC. Fairbairn et al. (1976) documented cannabinoid degradation rates at varying temperatures, Zamengo et al. (2019) studied changed under a variety of storage conditions, and Trofin et al. (2011) studied stability of select cannabinoids over a four year period. The consensus is that cannabinoids decrease over time during storage, which is accelerated by light or high temperatures (Fairbairn et al. 1976; Grafström et al. 2019; Lindholst 2010; Mazzetti et al. 2020; Zamengo 2019). However, our study contradicts the consensus because there was no degradation in total cannabinoids regardless of storage treatment. Perhaps the difference is that our study included the commercial canning process for postharvest storage. The canning process itself reduces the potential for gas exchange significantly and may have contributed to the preservation of cannabinoids. It is suggested that canning alone could be effective for maintaining HVCs with post-harvest storage times up to 76 days. Lindholst (2010) has also previously demonstrated the role of oxygen availability in reducing THC degradation rates of dried resin. It is also possible that more time was required to observe degradation, as some previous work would investigate degradation after years of storage versus weeks or months (Fairbairn et al. 1976; Zamengo et al. 2019). Previous studies have also frequently incorporated a smaller number of cannabinoids, and evaluating total cannabinoid dynamics was not feasible.

Most specific cannabinoids had no significant change over time or between storage treatments. The only cannabinoids that significant changed during storage included THCA, CBG, CBNA, CBC, THCV, and Δ^9^-THC. There are a few mechanisms that could explain increases in certain cannabinoids. One degradation pathway includes the oxidation of THC to CBN (Grafström et al. 2019). An increase in Δ^9^-THC for example, was observed for both treatments and contradicts work from Lindholst (2010) and Trofin et al. (2012) who reported Δ^9^-THC levels in dried resin decreased over time, as well as Turner et al. (1973), Fairbairn et al. (1976), and Zamengo et al. (2019) who all reported decreases at various rate ranges dependent on ambient storage temperature and light permanence of the storage container. Further, it has been well established that Δ^9^-THC can be synthesized through thermal decarboxylation of THCA (Tan et al. 2018; Tahir et al. 2021), and it is noteworthy that THCA was the only cannabinoid to decrease in our study. We propose that Δ^9^-THC accumulation in this our study is due to decarboxylation or oxidation of other cannabinoids or compounds.

CBG and THCV are the only cannabinoids to increase only under N_2_ MAP, which may represent a significant benefit to MAP in cannabis. CBG has anti-inflammatory and anti-pain characteristics (Nachnani et al. 2020). CBG has demonstrated effectiveness as a neuroprotectant to reduce severity of illnesses like Parkinsons disease or multiple sclerosis (Granja et al. 2012; Mammana et al. 2019). Meanwhile, THCV also has neuroprotective properties (Garcia et al. 2011) but has shown potential for management of obesity and diabetes (Abioye et al. 2020). Even though N_2_ MAP did not change total cannabinoid concentration during storage there is value in the accumulation of specific therapeutic compounds.

Failure to observe a significant decrease in levels of total terpene contrasts previous research. Bueno et al. (2020) reported a 51.6% decrease in terpenes for flower samples of similar terpene content after one month of storage. This provides further support for canning as a method of cannabis storage to increase retention of HVCs, irrespective of the gaseous composition in the container headspace.

Much like the cannabinoids, there may be value to maintenance or accumulation of specific terpenes and terpenoids. Approximately 85% of the terpenes/terpenoids in cannabis were comprised of β-myrcene, β-caryophyllene, limonene, α-humulene, and linalool, which was identical to Bueno et al. (2020). Of these major components, N_2_ MAP preserved β-myrcene and increased α-humulene while it decreased limonene. Myrcene is reported to have anti-inflammatory properties (Surendran et al. 2021), while α-humulene is reported to have anti-cancer properties (Chen et al. 2019). Each would also contribute to cannabis’ aroma, so preservation of these compounds may also maintain the expected aroma of fresh cannabis despite storage.

There was an overall trend that monoterpenes decreased while sesquiterpenes increased during storage. That trend was accentuated under N_2_ MAP, where cannabis stored under N_2_ MAP had a greater decrease in monoterpenes and increase in sesquiterpenes than atmospheric storage. Previous work in cannabis observed all terpenes decreasing (Bueno et al. 2020). However, there are examples in other species of terpenes increasing during storage, especially in cool temperatures. For example, monoterpenes decreased while sesquiterpenes increased in *Citrus junos* at −21 °C, 5 °C, and 20 °C (Njoroge et al. 1996). The exact mechanism remains unknown with respect to the decrease in monoterpenes versus increase in sesquiterpenes. There was no significant shift in terpenoids, which again might be due to oxygen limitation from canning.

There were three limitations in our study that could perhaps be improved upon in the future. The first limitation is that there is no measurement of HVCs prior to storage. This should not devalue the results or conclusions, as it is reasonable to assume that there should be no differences between storage treatments initially, as there would be no time for them to exert any effects. However, it makes it impossible to determine the extent of HVC preservation from 0 to 18 days. The second limitation is that the final storage time for analysis was roughly 2.5 months. This was an intentional choice based partially on Bueno et al. (2020) who investigated a storage time of 1.5 months, but it does limit us from predicting what other changes may have occurred. For instance, it is possible that N_2_ MAP may have preserved HVCs much longer, had we seen decreases in atmospheric storage beyond 2.5 months. The third limitation is a relatively small sample size, which was financially limited. However, random error was mitigated through the randomized block design and allowed for detection of significant differences with even small relative changes.

Random error was further reduced through the careful selection samples in this experiment. Previous research has shown that the chemical profile of cannabis will vary significantly based on both position and organ tissue (Bernstein et al. 2019a, Danziger and Bernstein 2021). Cannabinoid concentration tends to increase with height (Bernstein et al. 2019a; Danziger and Bernstein 2021). This relationship was strongly correlated with intercepted light and strengthened when increasing light interception, through pruning or defoliation, caused an increase in cannabinoid concentration in lower branches (Danziger and Bernstein 2021). Cannabinoid concentration also tends to be highest in flowers; there is a 50% and 90% decrease in cannabinoid concentration in inflorescent leaves and fan leaves, respectively, when compared to flowers (Bernstein et al. 2019a). Harvesting samples of equal height and from identical organs greatly decreased random error in the experiment and helped identify true differences.

## Conclusion

Total cannabinoids didn’t change through storage or MAP, but there were changes to specific cannabinoids. Canned cannabis decreased in THCA during storage, but increased in Δ^9^-THC, CBNA, and CBC. N_2_ MAP only increased concentrations of CBG and THCV compared to atmospheric storage. There may be some therapeutic benefit to having higher CBG and THCV content, though producers would have to determine if it was worth the added expense of N_2_. Total volatile terpene compounds also didn’t change through storage or MAP, but there was an overall decrease in monoterpenes and increase in sesquiterpenes under N_2_ MAP. Specifically, N_2_ MAP preserved β-myrcene, increased α-humulene, and decreased limonene compared to atmospheric storage.

The fact that volatile terpene compounds and cannabinoids didn’t degrade during storage is a novel finding that contradicts previous research. From a practical standpoint it provides reasonable evidence that canning cannabis for storage offers significant protection to HVCs, though there was no uncanned treatment for comparison. Future studies could compare the effectiveness of several commercial storage methods for preservation of HVCs, which may help standardize storage procedures to ensure a high value product.

## Supplementary Information

Supplementary Material 1.

## Data Availability

Data may be made available upon reasonable request.

## Abbreviations

Δ^8^-THC: Δ^8^-Tetrahydrocannabinol
Δ^9^-THC: Δ^9^-Tetrahydrocannabinol
CBC: Cannabichromene
CBCA: Cannabichromenic acid
CBD: Cannabidiol
CBDA: Cannabidiolic acid
CBDV: Cannabidivarin
CBDVA: Cannabidivarinic acid
CBG: Cannabigerol
CBGA: Cannabigerolic acid
CBL: Cannabicyclol
CBLA: Cannabicyclocic acid
CBN: Cannabinol
CBNA: Cannabinolic acid
GC-MS: Gas chromatography-mass spectrometry
HVC: High value compound
MAP: Modified atmospheric packaging
THCA: Tetrahydrocannabinolic acid
THCV: Tetrahydrocannabidivarin
THCVA: Tetrahydrocannabidivarinic acid
UPLC: Ultra performance liquid chromatography
