# Supplementary material for: Is nitrogen-modified atmosphere packaging a tool for retention of volatile terpenes and cannabinoids in stored Cannabis sativa inflorescence?
Source: J Cannabis Res. 2024 Dec 20;6:42. doi: 10.1186/s42238-024-00253-9 (PMC11660729; doi:10.1186/s42238-024-00253-9)
Supplement: Supplementary file 1 — Supplementary Material 1. [file 42238_2024_253_MOESM1_ESM.docx]

**Supplemental Tables**

**Supplemental Table I.** List of cannabinoids and their abbreviations analyzed for effect of MAP on storage degradation in cannabis. Cannabidiol and cannabicycloic acid were also analyzed but were below non-detectable limits.

| Compound | Abbreviation | CAS Registration Number |
| --- | --- | --- |
| Δ^9^-Tetrahydrocannabinol | Δ^9^-THC | 1972-08-3 |
| Δ^8^-Tetrahydrocannabinol | Δ^8^-THC | 5957-75-5 |
| Cannabichromene | CBC | 20675-51-8 |
| Cannabichromenic acid | CBCA | 20408-52-0 |
| Cannabidiolic acid | CBDA | 1244-58-2 |
| Cannabidivarin | CBDV | 24274-48-4 |
| Cannabidivarinic acid | CBDVA | 31932-13-5 |
| Cannabigerol | CBG | 25654-31-3 |
| Cannabigerolic acid | CBGA | 25555-57-1 |
| Cannabicyclol | CBL | 21366-63-2 |
| Cannabinol | CBN | 521-35-7 |
| Cannabinolic acid | CBNA | 2808-39-1 |
| Tetrahydrocannabinolic acid | THCA | 23978-85-0 |
| Tetrahydrocannabidivarin | THCV | 28172-17-0 |
| Tetrahydrocannabidivarinic acid | THCVA | 39986-26-0 |

**Supplemental Table II.** List of terpenoids analyzed for effect of MAP on storage degradation in cannabis. An additional 38 terpenoids were tested but were below non-detectable limits.

| **Terpene Name** | **Terpene Name** |
| --- | --- |
| 𝛼-Pinene | Fenchone |
| Terpinolene | *cis*-𝛽-Ocimene |
| Camphene | *trans*-𝛽-Ocimene |
| Linalool | Caryophyllene oxide |
| 𝛽-Pinene | Octyl acetate |
| 𝛽-Myrcene | Borneol |
| 𝛽-Caryophyllene | 𝛼-Terpineol |
| 𝛼-Humulene | 𝛼-Bisabolol |
| Limonene | Terpinen-4-ol |
| *trans*-Nerolidol | Geranyl acetate |
| Eucalyptol | 𝛽-Cedrene |
| Guaiol |  |

**Supplemental Table III.** Vanquish HPLC parameters for analysis of cannabinoids.

| Column | Ascentis Express 90 Å C18 15 cm x 2.1 mm x 2 µm |
| --- | --- |
| Column Temp | 30 °C |
| Mobile Phase A | 5 mM ammonium formate in water + 0.1% v/v formic acid |
| Mobile Phase B | 0.1% formic acid in acetonitrile |
| Flow Rate | 0.4 mL/min |
| Gradient | 0.0 min: 70% B 3.0 min: 90% B 5.0 min: 90% B 5.1 min: 98% B 6.0 min: 98% B 6.1 min: 70% B 8.0 min: 70% B |
| Detection | UV 228 nm |
| Injection | 25 μL |
| Elution Order | Ibuprofen, CBDVA, CBDV, CBDA, CBGA, CBG, CBD, THCV, THCVA, CBN, CBNA, Δ^9^-THC, Δ^8^-THC, CBL, CBC, THCA, CBCA, CBLA |

**Supplemental Table IV.** GC and injector parameters for analysis of terpenoids.

| Injection Volume (µL) | 1 |
| --- | --- |
| Liner | 4 mm Splitless Single Taper |
| Inlet (°C) | 220 |
| Inlet Module and Mode | SSL, Split |
| Carrier Gas (mL/min) | Helium (1.5) |
| Split Flow (mL/min) | 6 |
| Oven Temperature Program: | |
| Temperature 1 (°C) | 60 |
| Hold Time (min) | 0.5 |
| Temperature 2 (°C) | 130 |
| Rate (°C/min) | 50 |
| Hold Time (min) | 3 |
| Temperature 3 (°C) | 140 |
| Rate (°C/min) | 5 |
| Hold Time (min) | 0 |
| Temperature 4 (°C) | 250 |
| Rate (°C/min) | 22 |
| Hold Time (min) | 0 |
| Temperature 5 (°C) | 300 |
| Rate (°C/min) | 120 |
| Hold Time (min) | 2 |

**Supplemental Table V**. ISQ 7000 mass spectrometer parameters for analysis of terpenoids.

| Transfer Line (°C) | 250 |
| --- | --- |
| Ionization Type | EI |
| Ion Source (°C) | 300 |
| Acquisition Mode | SIM |
